# In Silico Structure‐Guided Design of Peptide Candidates Targeting γ‐Secretase Subunit Assembly

**DOI:** 10.1002/prot.70137

**Published:** 2026-04-02

**Authors:** Selcen Arı Yuka, Kübra Telli, Alper Yılmaz

**Affiliations:** ^1^ Department of Bioengineering Yildiz Technical University Istanbul Turkey; ^2^ Health Biotechnology Joint Research and Application Center of Excellence İstanbul Turkey; ^3^ Department of Molecular Biology and Genetics Istanbul Kultur University Istanbul Turkey; ^4^ Department of Molecular Biology and Genetics Yildiz Technical University Istanbul Turkey

**Keywords:** APH1, in silico peptide design, molecular docking, molecular dynamics simulations, notch signaling, peptide therapeutics, γ‐Secretase

## Abstract

The γ‐secretase complex is a membrane‐embedded protease essential for intramembrane cleavage of substrates such as Notch receptors and the amyloid precursor protein (APP), processes central to cancer progression and Alzheimer's disease (AD) pathology. However, catalytic inhibition of γ‐secretase disrupts multiple signaling pathways, resulting in dose‐limiting toxicities. In this study, we report a structure‐guided approach to generate peptides with binding and stability profiles that disrupt the assembly of γ‐secretase by targeting the interactions of Presenilin‐1 and Nicastrin with APH1. First, molecular docking was performed for 36 248 peptides of varying lengths to assess their affinity scores to the PS1 and NCT interaction regions of APH1. Peptides filtered based on their affinity scores and physicochemical properties were then subjected to global molecular docking. 50‐nanosecond molecular dynamics simulations and MM/PBSA analyses were performed on the top 10 potential candidates, identifying those with high dynamic interaction potential. Thus, seven γ‐secretase inhibitor candidates with favorable affinity scores capable of providing stable interactions and thereby having the potential to disrupt the APH1:PS1 assembly were identified. This approach, which overcomes the challenges of targeting the transmembrane catalytic domain, is based on the inhibition of subunit assembly and presents promising candidates for future experimental studies.

## 1 Introduction

The γ‐secretase complex is a multi‐subunit, intramembrane protease that orchestrates critical proteolytic events across a broad substrate spectrum, most notably the Notch receptors and the APP. These proteolytic processes are not merely mechanistic necessities but are functionally consequential, triggering signaling cascades that shape cellular differentiation, proliferation, and fate decisions [1, 2]. Dysregulation of γ‐secretase activity has therefore emerged as a central axis in the pathogenesis of both AD and multiple cancers, rendering this protease a high‐value yet elusive therapeutic target [3]. Structurally, γ‐secretase comprises four integral membrane proteins Presenilin (PS1/2), Nicastrin (NCT), APH1, and PEN‐2 assembled into a catalytically active configuration within the lipid bilayer [4]. Presenilin forms the enzymatic core of the complex and undergoes autoproteolysis to generate the active heterodimer [5]. In the nervous system, aberrant γ‐secretase‐mediated processing of APP generates amyloid‐β (Aβ) peptides, particularly Aβ42, which aggregate into plaques and drive neuroinflammation, synaptic dysfunction, and neurodegeneration characteristic of AD [6, 7]. Familial AD mutations in PSEN1, PSEN2, and APP further accentuate this cleavage imbalance [8]. Early pharmacologic interventions using broad γ‐secretase inhibitors (GSIs) showed promise in reducing Aβ production but failed clinically due to Notch‐related toxicities, highlighting the need for more precise modulation [3, 9]. In oncologic contexts, γ‐secretase activity facilitates the proteolytic release of the Notch intracellular domain (NICD), a transcriptional regulator that promotes tumorigenesis through enhanced cell proliferation, survival, and stemness [10, 11]. Overactive Notch signaling is a well‐established driver in T‐cell acute lymphoblastic leukemia, triple‐negative breast cancer, glioblastoma, and neuroendocrine tumors [12]. Presenilin facilitates NICD release, promoting proliferation and stemness, while NCT overexpression correlates with poor prognosis in breast and colon cancers [10]. Other subunits like APH1 and PEN‐2 contribute to receptor specificity and signal integration, respectively [13]. GSIs have shown promise in preclinical models of T‐cell leukemia, breast cancer, and glioma by inhibiting Notch signaling [1, 14]. However, clinical efficacy has been inconsistent, with challenges including subunit isoform diversity, off‐target protease activity, and compensatory oncogenic pathways [1, 15]. Furthermore, low‐dose GSI exposure has paradoxically enhanced Notch signaling, while some GSIs exhibit proteasome inhibition, further confounding their antitumor effects [16]. Taken together, the γ‐secretase Notch axis represents a biologically and therapeutically significant system, yet its complexity demands a nuanced approach. Ongoing efforts focus on achieving substrate‐ and context‐specific modulation to harness its therapeutic potential while minimizing systemic toxicity. To overcome these limitations, we present a rational, structure‐informed approach to design in silico‐engineered peptides that selectively disrupt γ‐secretase assembly by targeting APH1 a noncatalytic but essential scaffold subunit responsible for stabilizing the functional complex [17]. By interfering with APH1's interactions with PS1 and NCT, our peptide‐based strategy seeks to allosterically modulate the enzyme's activity, offering substrate‐sparing and potentially tissue‐specific inhibition [18]. Using atomic‐resolution models (PDB ID: 5A63) and computational docking pipelines (AutoGridFR, ADCP), we constructed peptide libraries enriched with interface‐critical residues (e.g., Gln, Phe, Tyr, Ile, Glu, Leu) and screened them for affinity, stability, and safety [19, 20]. The most promising candidates underwent global docking validation and molecular dynamics (MD) simulations to assess structural robustness [21, 22, 23]. Crucially, physicochemical and biological property filtering such as the instability index, allergenicity, and toxicity enabled the selection of therapeutically viable leads [24, 25, 26]. These peptides represent a new modality in modulating γ‐secretase activity without directly targeting its catalytic site, reducing systemic toxicity while retaining efficacy against disease‐specific pathways. This study proposes a novel strategy to selectively dismantle γ‐secretase functionality at the subunit interface level, potentially attenuating oncogenic Notch signaling and neurotoxic Aβ production without compromising essential physiological processes. The integration of peptide design, structural biology, and systems pharmacology paves the way for next‐generation γ‐secretase modulators with dual application in neurodegeneration and oncology.

## 2 Materials and Methods

### 2.1 Residue Extraction From Interfaces Between Subunits of Human γ‐Secretase Complex

The atomic coordinates of the human γ‐secretase complex (PDB ID: 5A63) were used to identify interaction interfaces involving the scaffold subunit APH1 with PS1 and NCT [17]. Structural analysis was conducted using PDBsum to extract critical residues at interfaces between subunits [27]. Key interaction residues identified for PS1 included Gln464, Phe465, Tyr466, and Ile467, while corresponding contact points on APH1 included Asn207, Tyr210, Ser160, and Gln83. NCT‐APH1 interactions were mediated primarily via Glu669, Glu37, Tyr41, and Leu662 (NCT) and Gly2, Asp140, Gly145, and Val146 (APH1).

### 2.2 Peptide Library Construction and Virtual Screening

Peptide libraries were designed to target APH1 interfaces using six critical amino acids (Gln, Phe, Tyr, Ile, Glu, Leu) based on their observed involvement in stabilizing APH1 interactions. An initial combinatorial library of 7776 pentapeptides was generated. Peptide–protein docking was performed separately at the APH1‐PS1 and APH1‐NCT interfaces using AutoDock CrankPep (ADCP v1.0) [18], after preparing the affinity maps of binding regions on APH1 using AutoGridFR [28]. Each docking simulation utilized 5 000 000 scoring evaluations across 30 independent searches. The median affinity scores of the top 20 poses were used to rank peptide performance. Top‐scoring pentapeptides were subsequently expanded into hexapeptides by permutation and filtered through iterative virtual screening. Libraries of 6–10 amino acid peptides were evaluated using the same docking protocol. A total library of 36 248 peptides of different amino acid lengths was analyzed. All screening tasks were parallelized and executed on TÜBİTAK ULAKBİM High Performance and Grid Computing Center‐Turkish National Science e‐Infrastructure (TRUBA)'s Arf cluster using GNU Parallel and custom SLURM scripts [29].

### 2.3 Physicochemical and Safety Profiling

The peptides were analyzed for physicochemical properties including net charge, isoelectric point, instability index, and Boman index using the Peptides R package (v2.4.1) [19]. Allergenicity and toxicity predictions were performed using AllerCatPro2 and ToxinPred3, respectively [20, 30]. Candidate peptides were retained if they satisfied the following criteria: |charge| < 2, instability index < 40, Boman index between 0 and 2.48, and predicted nontoxic and nonallergenic profiles [24, 25, 31, 32].

### 2.4 Global Peptide–Protein Docking

Ten lead peptides were selected for further validation via global docking using CABS‐dock and HPEPDOCK [23, 33]. No predefined binding sites or flexible residue constraints were applied. Each docking platform generated 10 cluster models, evaluated using RMSD (CABS‐dock) and docking scores (HPEPDOCK). Predicted binding modes were compared to assess binding site specificity and spatial convergence of docking poses.

### 2.5 MD Simulations and MM/PBSA Analysis

Peptide‐APH1 complexes were subjected to MD simulations to evaluate complex stability. Structures were preprocessed using UCSF Chimera (v1.16) [34]. All simulations were performed with GROMACS 2024.1 [21] using the OPLS‐AA force field. Systems were solvated in a cubic box with SPC216 water molecules and neutralized to 0.15 M NaCl. Following energy minimization, 1 ns NVT and 3 ns NPT equilibration phases were conducted, prior to 50 ns MD production runs at 300 K. The analysis focused on the backbone root‐mean‐square deviation (RMSD), root‐mean‐square fluctuation (RMSF), hydrophobic interactions, hydrogen bonds, and radius of gyration was performed, and the stability of peptide: protein interactions was evaluated according to these metrics.

To calculate the binding free energies of peptide–protein complexes, MD simulations were first performed, followed by Molecular Mechanics Poisson–Boltzmann Surface Area (MM/PBSA) analysis to determine the binding energy and perform decomposition analysis using the gmx_MMPBSA v1.6.3 tool [35]. The analysis was performed on frames extracted from 30 to 50 ns production range of the selected MD trajectory after conformational stability was ensured with backbone RMSD convergence checks [36]. A sampling interval of 20 frames was applied to ensure representative conformational coverage. In the Poisson–Boltzmann calculations, the ionic strength was set to 0.15 M, and the internal and external dielectric constants were assigned values of 2.0 and 78.5, respectively. For molecular surface area calculations, a probe radius of 1.4 Å was used, and Van der Waals surface radii were assigned according to the AMBER force field parameters. To address the critical role of entropic contributions in flexible peptide systems, the Interaction Entropy (IE) method was employed. To ensure statistical convergence and minimize noise arising from potential conformational transitions, the parameter ie_segment = 50 was used. To provide a detailed breakdown of individual amino acid contributions, an energy decomposition analysis (idecomp = 2) per residue was performed.

## 3 Results

### 3.1 Virtual Screening Identifies High‐Affinity APH1‐Binding Peptides

A combinatorial virtual screening campaign was conducted using peptide libraries targeting two key interfaces within the γ‐secretase complex: APH1:PS1 and APH1:NCT. Initial local docking of 7776 pentapeptides yielded several candidates with favorable median affinity scores below −9.20 and −7.95 kcal/mol across PS1 and NCT subunits interaction sites. Peptides containing Phenylalanine (F), Tyrosine (Y), Glutamic Acid (E), and Glutamine (Q) exhibited the best median affinity scores within the NCT and PS1 binding regions, suggesting the involvement of hydrophobic‐aromatic and ionic/polar interactions at both interfaces. The expansion of top‐ranking pentapeptides predicted to interact with both PS1 and NCT subunit interaction sites into hexapeptides, followed by iterative docking, revealed sequence‐specific enhancements in binding affinities. Notably, some hexapeptides exhibited median affinity scores as low as −21.00 kcal/mol at the PS1 interface and −17.25 kcal/mol at the NCT interface. The iterative process was conducted up to 10‐mer long peptides and the distributions of the median affinity scores for each set of peptide lengths for NCT and PS1 were computed (Figure S1). When the overall landscape is examined, a systematic enrichment of the peptide population with higher predicted affinities is observed; this progressive shift in the mean docking scores demonstrates that each iteration effectively narrows the search space toward stronger candidates. This iterative enhancement resulted in peptide sequences that exhibited better affinity for both sites with increasing peptide length, while it was generally observed that candidates exhibited higher affinity for the PS1 binding site of APH1 compared to the NCT site (Figure S1). Beyond the affinity scores, the physicochemical properties of the peptides were also investigated for peptide selection. An appropriate instability index (< 40) and a Boman index threshold in the range of 0–2.48 were applied and peptides with high net charge (|charge| ≥ 2) were excluded during primary filtering [24, 25, 31, 32]. This primary filtering resulted in 2120 peptides with various lengths (Table S1). To establish a biologically relevant threshold for candidate selection, the C‐terminal sequence of PS1 (LAFHQFYI, an 8‐aa fragment) was utilized as a reference ligand. As a result of molecular docking between APH1 and LAFHQFYI, the best model showed an affinity score of −18.8 kcal/mol. This value was subsequently adopted as a reference for screening the designed peptide library. Therefore, 241 peptides with affinity score lower than −19 kcal/mol were retrieved from the set of peptides showing favorable physicochemical properties and these peptides were found to have nonallergenic and nontoxic profiles. Out of 241 peptides, 10 peptides were picked and subjected to further analysis (Table 1). The general profile of these sequences showed a high proportion of sequences containing aromatic and polar amino acids.

**TABLE 1:** Engineered peptides for inhibition of APH1:PS1 interaction and their characteristics.

Peptide	No	Affinity score (NCT)	Affinity score (PS1)	Charge	pI	Stability	Boman index	CABS‐Dock RMSD	HPEPDOCK score
YQYQYLYFYY	1	−18.5	−23.2	0	6.1	38.86	0.402	0.92	−266.57
YYLYYQYYFE	2	−19.5	−23.05	−1	3.8	38.48	0.529	3.17	−266.878
YQYFQYLYYF	3	−18.4	−22.95	0	6.1	6.26	0.09	0.86	−270.864
YQYLYFYYQY	4	−18.6	−22.9	0	6.1	38.86	0.402	2.17	−275.435
YQYLYFYYEF	5	−18.7	−22.85	−1	3.8	38.86	0.217	2.65	−270.309
YFQYLYYFEY	6	−19.45	−22.85	−1	3.8	13.8	0.217	4.46	−256.333
FQYYLYYQYY	7	−18.6	−22.85	0	6.1	30.94	0.402	1.48	−276.258
QYYLYYYE	8	−16.75	−19.2	−1	3.8	36.17	1.01625	1.76	−239.726
EYQYYLYF	9	−16.5	−19.2	−1	3.8	14.75	0.62625	3.69	−234.760
EYQYIYYY	10	−16.6	−19.2	−1	3.8	30.18	1.01625	1.03	−233.316

### 3.2 Global Docking Validates Interface‐Specific Binding

To reaffirm the interaction of the peptides designed with the ADCP local molecular docking virtual screening workflow with the binding sites of the target protein, analysis was performed with CABS‐dock and HPEPDOCK global molecular docking methods. According to the analysis performed with CABS‐Dock, Peptides 1, 3, 7, 8, and 10 were stable (RMSD < 2), Peptides 2, 4, 5, and 9 were moderately stable (2 < RMSD < 4), and Peptide 6 was unstable (4 < RMSD) [33]. HPEPDOCK rankings similarly corroborated binding consistency, with top‐scoring clusters mainly aligning to the PS1‐facing clefts of APH1 without extraneous off‐site interactions. These findings indicate that the majority of selected peptides retained binding specificity even in the absence of spatial constraints, suggesting intrinsic affinity for the APH1 surface topography defined by PS1 and NCT interfaces. Moreover, the global docking results of the 8‐aa sequence (LAFHQFYI) extracted from the C terminus of PS1 showed an RMSD value of 0.77 in CABS‐Dock and an HPEPDOCK score of −233.693, supporting the comparable potential of the peptides engineered by virtual screening.

**FIGURE 1:**
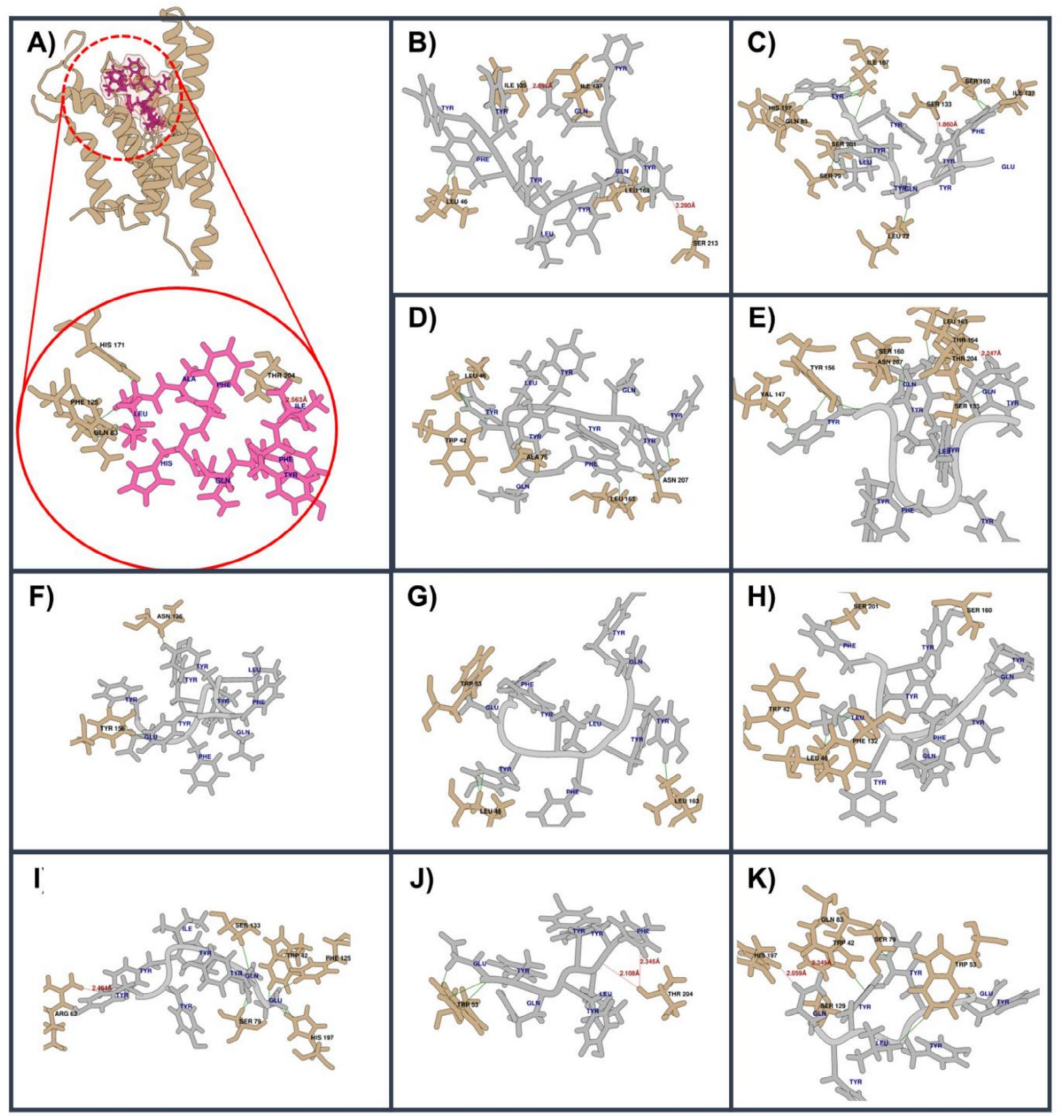
Peptide:APH1 interaction interfaces. (A) 8‐aa C‐terminal peptide of PS1 interacted with APH1. (B–K) represents hydrogen bonds (red) and contacts (green) at the interfaces of Peptides 1–10, respectively.

The PS1‐C terminal interaction with APH1 in the complex involved a 2.563 Å length hydrogen bond through Thr204, as well as mostly hydrophobic and polar interactions with residues such as Phe125, His171, and Gln83 (Figure 1A). The interaction profile between Peptide 1 and APH1 was characterized by prominent hydrophobic contacts and π–π stacking interactions. These interactions predominantly involved APH1 residues such as Leu46, Ile135, Ile137, and Leu163. Additionally, specific hydrogen bonds were observed between Gln2 of Peptide 1 and Ile135 of APH1 (2.592 Å), as well as between Tyr3 and Ser213 (2.280 Å), as illustrated in Figure 1B. In the APH1 interaction interface of Peptide 2, a 1.860 Å‐length hydrogen bond was found with Ser133. The interactions revealed that several amino acids may contribute especially in hydrophobic contacts and aromatic stacking, such as Ile167, His197, and Ser160 (Figure 1C). Although no hydrogen bonds were identified at the Peptide 3:APH1 interface, contact analysis suggested the involvement of recurring residues, such as Asn207, Trp42, Leu46, and Leu163, in stabilizing the interaction through hydrophobic contacts and π–π stacking (Figure 1D).

Peptide 4, similar to the PS1‐C terminal domain, was prone to hydrogen bonding with Thr204 (2.247 Å). Moreover, this peptide formed a similar interaction pattern with residues Tyr156, Asn207, Leu163, Thr204, Thr164, and Ser160 as the PS1‐C terminal domain with the potential for aromatic stacking and hydrophobic interaction (Figure 1E). Peptide 5 binds to the APH1 interface primarily by contacting residues Tyr156 and Asn136; Peptide 6 is characterized by contacts primarily via Trp53, Leu46, and Leu163; however, it is noteworthy that neither peptide forms hydrogen bonds (Figure 1F,G). The Peptide 7:APH1 interface exhibited a dense contact profile despite the absence of hydrogen bonds. Specific residues such as Trp42, Ser160, Leu46, and Ser201 were prominently featured and displayed interaction patterns comparable to those observed in complexes formed with other peptides (Figure 1H). Notably, the interaction between Peptide 8 and APH1 was characterized by a hydrogen bond of 2.482 Å in length formed with Arg62. Furthermore, the interaction interface of APH1 was predominantly defined by key residues including His197, Trp42, Ser79, Ser133, and Phe125, which contributed to a variety of noncovalent interactions such as hydrogen bonding, electrostatic forces, π–π stacking, and polar contacts (Figure 1I). The Peptide 9:APH1 complex exhibited potential hydrogen bonding between Leu6 and Phe8 of the peptide and Thr204 of APH1. In addition, interactions with Trp53 have also been observed (Figure 1J). Peptide 10 demonstrated hydrogen bonding between the Gln residue of the peptide and Gln83 and His197 of APH1, with bond lengths of 2.349 Å and 2.059 Å, respectively. Additionally, interactions were predominantly concentrated at residues Trp42, Ser129, Ser79, Trp53, and His197. While the interaction profile encompassed features similar to those observed with other peptides, including aromatic stacking and hydrophobic contacts, a distinct affinity for certain residues was noted, differentiating it from the interaction profiles of other peptides (Figure 1K). Overall, the interaction profiles of the designed peptides were strikingly similar, with each engaging APH1 through comparable noncovalent interactions, including hydrogen bonds, hydrophobic interactions, and aromatic stacking. However, certain peptides exhibited distinct differences in their binding sites and the specific amino acid residues involved.

### 3.3 MD Reveal Peptide‐APH1 Complex Stability

**FIGURE 2:**
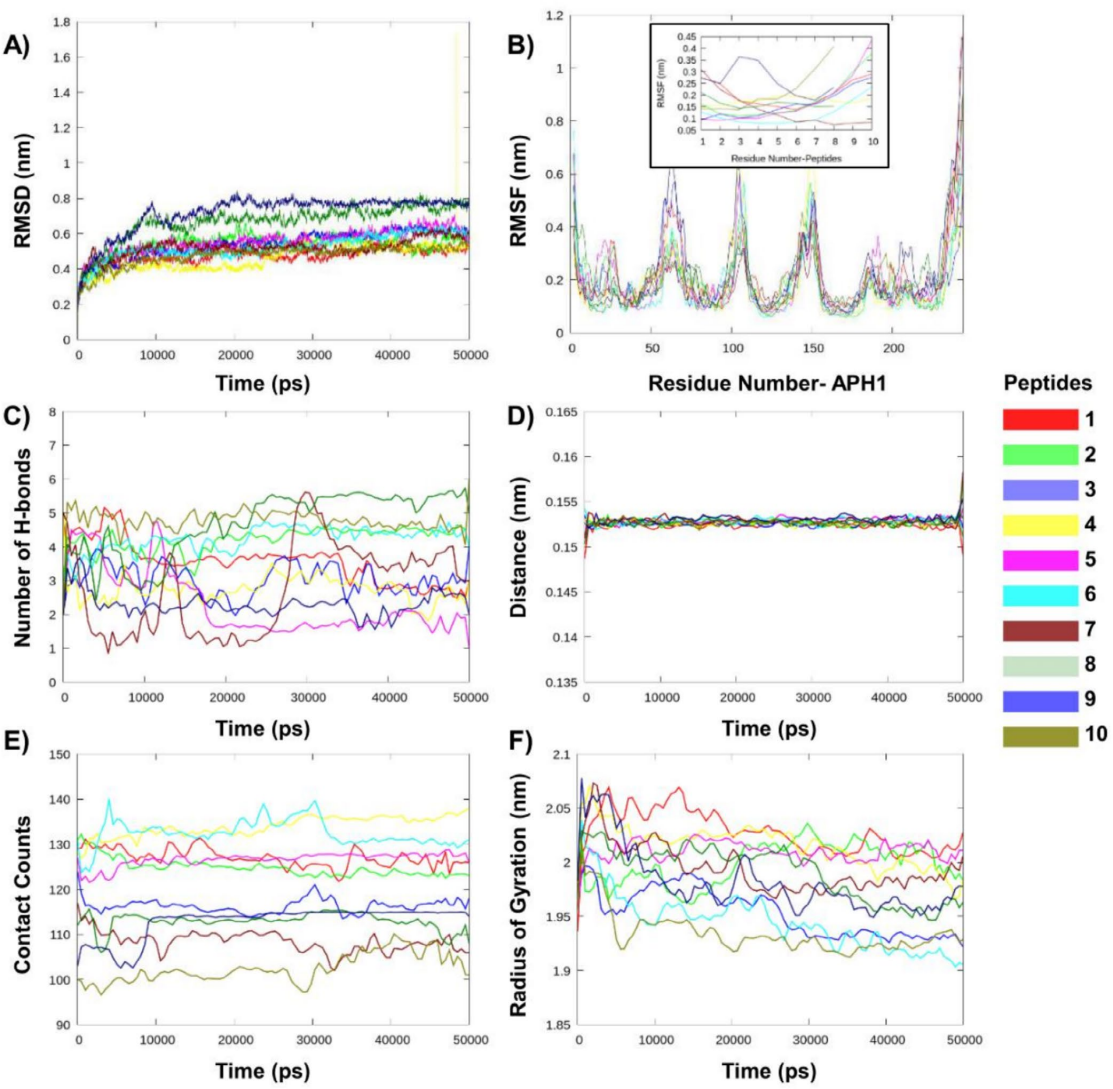
Comprehensive structural and interaction analyses of 10 protein–peptide complexes throughout 50 ns MD simulations. (A) RMSD profile of complexes, backbone after lsq fit to Backbone. (B) Per‐residue RMSF profiles of both protein and peptides. (C) Hydrogen bond profiles (D) The hydrophobic distance profiles and (E) contact counts in the Peptide:APH1 interfaces. (F) Radius of gyration (*R*_g_).

The 10 lead peptides were subjected to 50 ns of all‐atom MD simulations with APH1 to evaluate structural stability, flexibility, compactness, and interactions. The RMSD profile, which was constructed from backbone atoms, showed that the largest proportion of complexes reached equilibrium in the first 10 ns of the simulation and stabilized in the range of approximately 0.4–0.8 nm (Figure 2A). These RMSD profiles showed that the peptides conformed to the upper limit of clustering for evaluating whether they were in the binding region of APH1 [37, 38]. The dramatic peak (~1.8 nm) observed in the Peptide 4:APH1 complex in the last phase of the simulation may indicate a critical issue, such as a significant conformational change or the peptide detaching from the binding site. Moreover, in the RMSF profiles of the complexes, the fluctuations on APH1 are generally consistent with the characteristic features of the protein and are concentrated in the N‐ and C‐terminal regions or in flexible loop structures (Figure 2B).

When evaluated in terms of hydrogen bonds, dramatic changes are observed in Peptides 5 and 7, while in other peptides, the number of hydrogen bonds generally varies within a narrow range (Figure 2C). Hydrophobic contact count and distance analysis of the interfaces of APH1 complexes of peptides reveal the number and consistency of hydrophobic interactions (Figure 2D,E). Peptide 10 exhibits the least contact profile among the complexes, while Peptide 6 has a prominent hydrophobic interaction profile (Figure 2E). Hydrophobic distance profiles at a range of ~0.145–0.160 nm in all complexes showed that a certain structure was found between the hydrophobic surfaces and that the interactions between these regions were stable (Figure 2D). In all the complexes, *R*_g_ values remained in the range of ~1.95–2.05 nm throughout the simulation, and the structural integrity was maintained, especially from 20 ns onwards, with the preservation of three‐dimensional folding patterns and compact structure (Figure 2F). Overall, the designed peptides showed favorable RMSD and *R*_g_ profiles as well as a promising RMSF landscape. In addition, hydrophobic contact number and distance analyses showing the nature and consistency of binding indicated the potential of the designed peptides.

**FIGURE 3:**
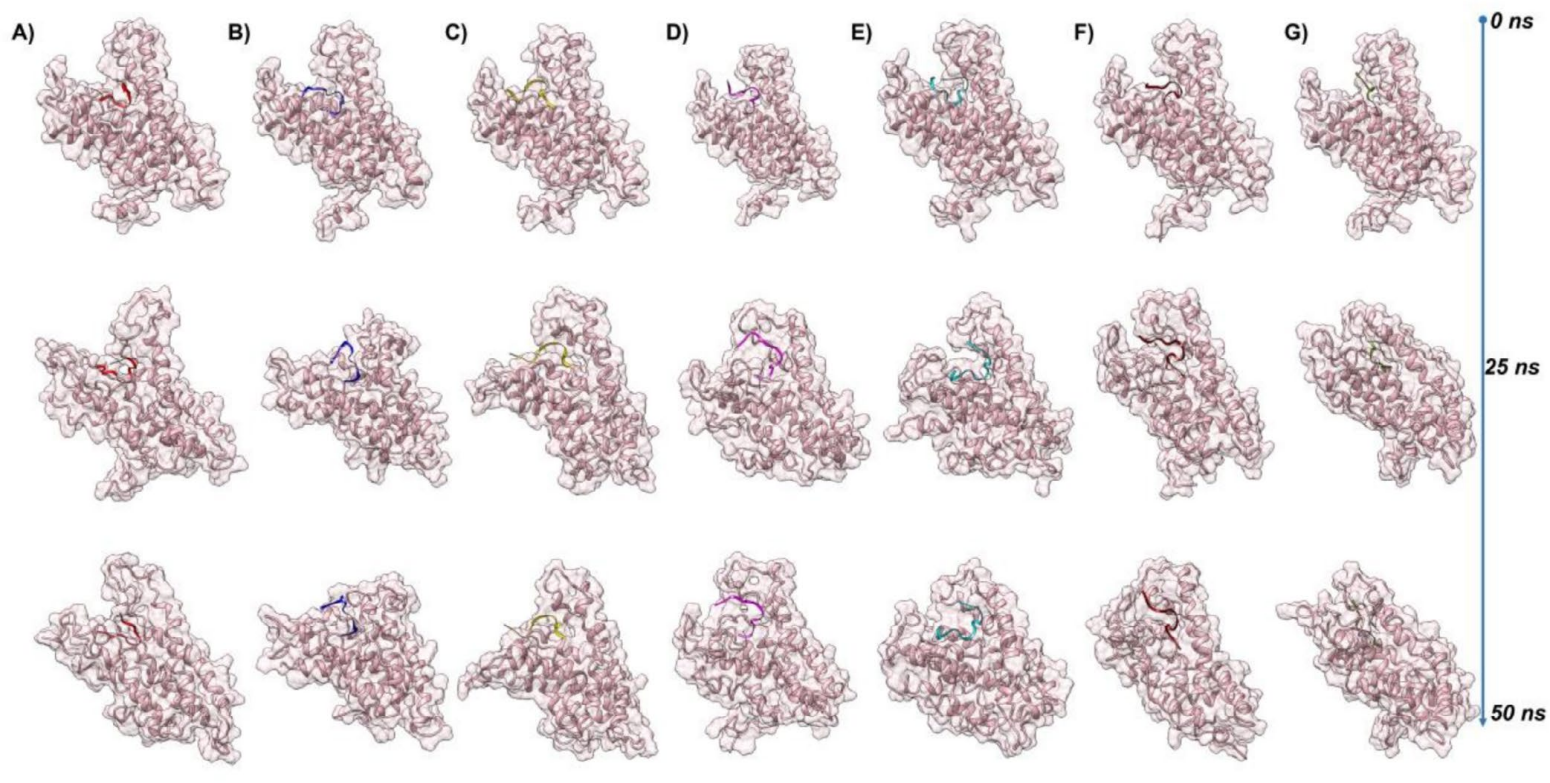
General view of the conformational changes and binding of peptides at time points 0, 25, and 50 ns from MD simulations of APH1 interactions of peptides. Each panel (A–G) represents complexes involving Peptides 1, 3, 4, 5, 6, 7, and 8 respectively.

As a result of the stability and binding analysis of these complexes, the potential of the interactions of Peptides 1, 3, 4, 5, 6, 7, and 8 was particularly noteworthy. The structural changes of these peptides were analyzed at 0, 25 and 50 ns time points (Figure 3). Initially, all peptides were bound to the protein surface, exhibiting a more regular and favorable conformation, whereas at 25 ns, some structural changes were observed in the position of the peptides and the protein structure in the binding site. It is likely that these changes, as assessed by the RMSD profiles, were characterized by a more stable complex. This stabilized conformation persisted at 50 ns, indicating that peptides continue to interact in the PS1‐interaction cavity of APH1 even in the presence of conformational changes in the complex. More specifically, Peptides 1, 5, 6, 7, and partially Peptide 8 showed a more buried and stabilized conformation at the peptide binding site, suggesting local induced fit mechanisms (Figure 3A,D–G) [39]. Among them, the minimal change in APH1 interactions of Peptide 1 indicates an optimal binding potency from the initial binding (Figure 3A). In contrast, Peptides 3 and 4 induced a relatively open conformation at the APH1 binding site (Figure 3B,C).

### 3.4 Molecular Mechanics/Poisson–Boltzmann Surface Area (MM/PBSA) Analysis

Following the MD simulations, the ΔTOTAL, Δ*G*_binding_ energy, and energy components were comprehensively analyzed by MM/PBSA calculations. While all peptides exhibited negative binding energies, indicating that protein binding is thermodynamically favorable and stable, the top candidates for overall Δ*G*_binding_ energy among the complexes were Peptides 1, 7, 4, 3, and 8, respectively (Table 2). Notably, Peptide 1 showed the highest binding affinity with a Δ*G*_binding_ value of −61.36 ± 12.16 kcal/mol, followed by Peptide 7 (−45.00 ± 8.72 kcal/mol).

**TABLE 2:** Binding free energy analysis and comparison of protein–ligand complexes by energy components with MM/PBSA.

Peptide	Δ*E*_vdW_	*ΔE*_elec_	Δ*E*_PB_	Δ*G*_GAS_	Δ*G*_SOLV_	ΔTotal	Δ*G*_binding_
1	−121.64 ± 3.32	−86.69 ± 0.59	108.89 ± 1.90	−91.71 ± 1.96	110.17 ± 1.07	−80.98 ± 4.47	−61.36 ± 12.16
2	−89.98 ± 1.88	−30.48 ± 4.36	56.16 ± 1.39	−120.45 ± 4.74	88.24 ± 1.60	−32.22 ± 5.01	−20.60 ± 7.19
3	−96.66 ± 3.03	−23.15 ± 3.36	47.38 ± 2.60	−119.81 ± 4.53	71.16 ± 3.12	−48.65 ± 5.50	−38.12 ± 4.57
4	−101.02 ± 2.23	−32.87 ± 3.35	57.44 ± 1.43	−133.90 ± 4.02	78.03 ± 2.13	−55.87 ± 4.55	−38.63 ± 7.22
5	−101.29 ± 3.39	−40.69 ± 0.65	74.32 ± 0.66	−141.98 ± 3.45	96.41 ± 2.14	−45.57 ± 4.06	−27.70 ± 6.88
6	−97.00 ± 2.76	−25.68 ± 5.39	48.01 ± 4.56	−122.68 ± 6.05	71.19 ± 5.17	−51.49 ± 7.96	−26.96 ± 5.90
7	−108.47 ± 2.48	−85.14 ± 3.62	107.63 ± 2.93	−193.61 ± 4.39	130.02 ± 3.05	−63.59 ± 5.34	−45.00 ± 8.72
8	−90.78 ± 2.03	−51.69 ± 3.80	67.71 ± 2.18	−142.47 ± 4.30	96.19 ± 2.42	−46.28 ± 4.94	−30.55 ± 6.56
9	−78.60 ± 2.25	−23.51 ± 5.28	46.75 ± 2.73	−102.11 ± 5.74	70.43 ± 2.74	−31.68 ± 6.36	−18.87 ± 5.90
10	−69.01 ± 2.26	−7.05 ± 3.79	25.13 ± 3.57	−76.06 ± 4.42	48.09 ± 3.69	−27.97 ± 5.76	−9.78 ± 6.06

*Note:* Δ*G*_binding_ values were calculated after Interaction Entropy (IE) approximation. All energy components are reported in kcal/mol.

A comprehensive analysis of the components of these binding energies revealed that Van der Waals and electrostatic interactions were particularly prominent in peptide:APH1 complexes. More specifically, the most prominent Van der Waals energies of −121.64 ± 3.32, −108.47 ± 2.48, and −101.29 ± 3.39 kcal/mol were noted in Peptides 1, 7, and 5, respectively. A similar profile was observed for the electrostatic interactions, but the electrostatic interactions in Peptide 8 were quite remarkable. It should also be pointed out that the polar solvent environment partially inhibits binding in Peptide:APH1 interactions, as expected from the system where solvent was applied in polar water environment. Δ*G*_GAS_ indicated that the binding energy in the gas phase enhances binding. In general, the bulk of the binding energy was due to Van der Waals and electrostatic interactions, while polar solvation energy partially inhibited binding, and nonpolar solvation and gas phase energies favored stability.

**FIGURE 4:**
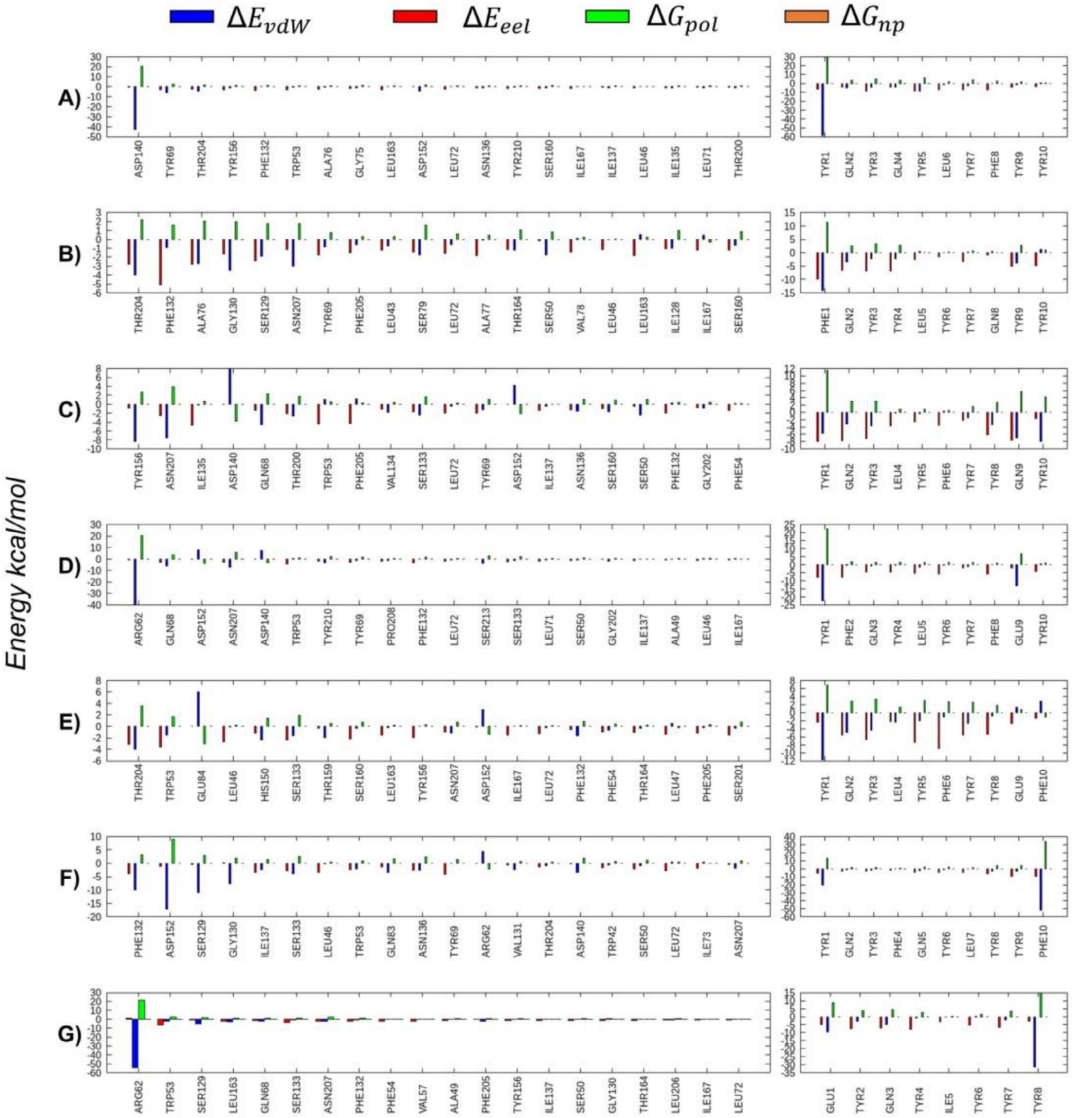
Per‐residue MM/PBSA free energy decomposition analysis of peptide–protein interactions. *ΔE*_vdW_: Van der Waals, *ΔE*_elec_: Electrostatic, *ΔG*_pol_: Polar solvation, and *ΔG*_np_: Nonpolar solvation are shown for the interactions between the protein and the peptides: (A) Peptide 1, (B) Peptide 3, (C) Peptide4, (D) Peptide 5, (E) Peptide 6, (F) Peptide 7, and (G) Peptide 8. Each panel highlights contributing residues at the protein–peptide interface. Residues on the receptor were ranked according to absolute total energy change, with the 20 highest residues displayed.

Decomposition analysis of the binding energies of Peptides except 2, 9, and 10 in the complexes revealed that each peptide exhibited dynamic and unique binding properties. Interestingly, the strong binding in the Peptide 1:APH1 complex was characterized by the energetic contribution of APH1‐Asp140 and Peptide 1‐Tyr1 residues (Figure 4A). On the other hand, in interactions with Peptide 3, APH1 was observed to exert electrostatic and Van der Waals interactions with residues such as Thr204, Phe132, Ala76, Gly130, Ser129, and Asn207. From the peptide side, the interactions were balanced and even favored the two terminals in particular (Figure 4B). The energy decomposition of Peptide 4 similarly included a variety of diverse energy components contributed by various residues, but interestingly residues Asp140 and 152 did not display favorable in terms of Van der Waals forces. For the peptide, a balanced and homogeneous energy distribution was observed (Figure 4C).

Similar to Peptide 1 in context of energy decomposition, one residue was also prominent in the interactions of Peptide 5 (Figure 4D). However, on the peptide side, the contribution of Tyr1 and Glu9 in terms of Van der Waals forces was critical. In the Peptide 6:APH1 interactions, various residues at the interface of the complex contributed to binding in varying proportions and exhibited a more homogeneous energy partitioning (Figure 4E). Although the effect of other residues on the interactions of Peptide 7 is significant, especially residue Phe10, contributed to an efficient energy partition in binding. On the APH1 side, Phe132, Asp152, Ser129, and Gly130 showed the most critical interactions (Figure 4F). In Peptide 8 interactions, similar to Peptide 5, APH1‐Arg62, and Peptide 8‐Tyr8 were prominent in energy decomposition, while the peptide also contributed to binding via Glu1, Tyr2‐4, and Gln3 (Figure 4G).

## 4 Discussion

Targeting the γ‐secretase complex has long presented a paradox in therapeutic design: its broad physiological roles render it essential, yet its dysregulation is directly implicated in the pathogenesis of AD and numerous cancers [1, 40]. In this study, we demonstrate a novel and selective strategy to modulate γ‐secretase function by disrupting its structural integrity at the subunit level specifically by targeting the APH1 scaffold protein with rationally designed peptides. By focusing on subunit assembly rather than catalytic inhibition, this interface‐driven approach offers an alternative route to overcoming dose‐limiting toxicities that arise from indiscriminate suppression of γ‐secretase activity [3, 9]. This approach was conceptualized to disrupt γ‐secretase assembly by targeting APH1‐mediated interactions with the PS1 and NCT subunits; however, local docking‐based virtual screening indicated that the PS1‐facing interface of APH1 was more favorable than the NCT interface in terms of predicted binding affinity. Moreover, unsupervised global blind docking analysis of candidates selected based on biophysical filters including stability, Boman index, and in silico toxicity and allergenicity screening also further corroborated this finding [24, 25, 30, 31, 32].

Naturally, spontaneous molecular docking‐based analysis of molecules as flexible as peptides is not solely adequate for a deep understanding of their dynamic interactions. Therefore, the dynamic properties of the interactions of the candidate peptides with the APH1 protein were evaluated by 50 ns MD simulations and further MM/PBSA analyses. Both the overall time‐dependent RMSD, residue‐based RMSF profiles, and *R*_g_ revealed the stability and consistency of the interactions of the peptide candidates [37, 38].

In MM/PBSA analysis, all peptides were found to be thermodynamically favorable (in the range from −27.97 ± 5.76 to −80.98 ± 4.47 kcal/mol). In general, most of the binding energy was attributed to Van der Waals and electrostatic interactions, but, as expected, polar solvation energy partially constrained binding. Peptide:APH1 binding energy decomposition analysis revealed that peptides exhibit dynamic and unique binding properties. For example, the strong binding in the Peptide 1:APH1 complex was characterized by energy contributions between Asp140 of APH1 and Tyr1 residues of the peptide. Collectively, all MD simulation results and MM/PBSA results together revealed that Peptides 1 (YQYQYLYFYY), 3 (YQYFQYLYYF), 4 (YQYLYFYYQY), 5 (YQYLYFYYEF), 6 (YFQYLYYFEY), 7 (FQYYLYYQYY), and 8 (QYYLYYYE) could be used for therapeutic applications for γ‐secretase inhibition.

This work departs from traditional γ‐secretase inhibitor development in several key respects. First, rather than targeting the catalytic site of Presenilin, whose inhibition indiscriminately blocks all substrate processing, including Notch and APP, we exploit subunit interaction surfaces to selectively interfere with complex assembly [6, 14]. By focusing on APH1, which lacks enzymatic activity but is essential for structural cohesion, we introduce a means to destabilize the γ‐secretase complex in a context‐sensitive manner. This is particularly relevant in cancer, where γ‐secretase activity drives oncogenic Notch signaling [11, 41], and in AD, where it governs amyloidogenic APP cleavage [7]. The APH1‐focused strategy circumvents the toxicities observed in past clinical trials of broad GSIs, offering a refined therapeutic axis with potentially fewer off‐target effects [9]. Moreover, this is the first report, to our knowledge, that computationally engineers short peptide inhibitors against APH1 using atomic‐resolution structural data, local interface analysis, and a multi‐tiered filtering and validation workflow [17, 18]. While recent efforts in peptide drug discovery have gained traction [42], few have been directed at membrane‐embedded protein–protein interactions, largely due to their structural inaccessibility. Our study demonstrates that interface‐specific binding sites can be successfully targeted, provided that peptide design is informed by subunit architecture and refined through modern simulation tools.

Our results provide a comprehensive in silico framework for identifying short peptide candidates capable of inhibiting the assembly of the γ‐secretase complex via APH1 targeting. However, there are certainly some limitations. First, although the γ‐secretase complex consists of transmembrane subunits, the assembly of the enzyme can occur in many cellular compartments that exhibit radical differences in terms of biochemical and lipid composition, such as the ER, Golgi apparatus, endosomes, and lysosomes [43, 44]. In this approach targeting enzyme assembly, membrane dynamics have been ignored due to the lack of a consensus membrane model that accurately reflects the functional nature of the enzyme. Specifically, the focus has been on peptide–protein interaction interfaces, and the dynamics of transmembrane units or membrane‐derived allosteric effects have been excluded. In future studies, the design of system membrane components may provide new perspectives for the approach; however, the fact that such comprehensive systems require specific parameterization and high infrastructure costs should not be underestimated. On the other hand, since all findings were obtained from computational modeling, experimental validation is still needed. Future studies should include in vitro binding assays of these candidate peptides, cellular studies evaluating γ‐secretase activity and downstream Notch/APP signaling, and ultimately assessment of pharmacokinetics, stability, and blood–brain barrier permeability. Peptidomimetic optimization, chemical stabilization, or delivery via nanoparticle systems may be required for translational efficacy [45]. Future work should also explore the tissue‐specific expression and isoform diversity of γ‐secretase subunits to tailor interventions across disease contexts. For example, cancer subtypes with elevated NCT or Presenilin variants may be especially sensitive to APH1‐targeted disruption [10]. Such studies will be essential for converting these computationally identified candidates into feasible therapeutics for Alzheimer's disease and cancer.

## Author Contributions

**Selcen Arı Yuka:** methodology, conceptualization, formal analysis, validation, writing – review and editing, writing – original draft, data curation, and investigation. **Kübra Telli:** methodology, conceptualization, writing – review and editing, investigation, and writing – original draft. **Alper Yılmaz:** methodology, supervision, and formal analysis.

## Funding

The authors have nothing to report.

## Conflicts of Interest

The authors declare no conflicts of interest.

## Supporting information


**Figure S1:** Distribution of energies according to molecular docking results of peptide sets with NCT and PS1 binding sites of APH1. Energy values are provided as the median affinity scores of the top 20 models in each interaction.
**Table S1:** Distribution by length of peptides with favorable physicochemical properties.

## Data Availability

The data that support the findings of this study are available from the corresponding author upon reasonable request.
