# Supplementary material for: In Silico Structure‐Guided Design of Peptide Candidates Targeting γ‐Secretase Subunit Assembly
Source: Proteins. 2026 Apr 2;94(8):1518–28. doi: 10.1002/prot.70137 (PMC13327434; doi:10.1002/prot.70137)
Supplement: Supplementary file 1 — Figure S1: Distribution of energies according to molecular docking results of peptide sets with NCT and PS1 binding sites of APH1. Energy values are provided as the median affinity scores of the top 20 models in each interaction. Table S1: Distribution by length of peptides with favorable physicochemical properties. [file PROT-94-1518-s001.docx]

**In Silico Structure-Guided Design of Peptide Candidates Targeting γ-Secretase Subunit Assembly**

**Supplementary Materials**

**
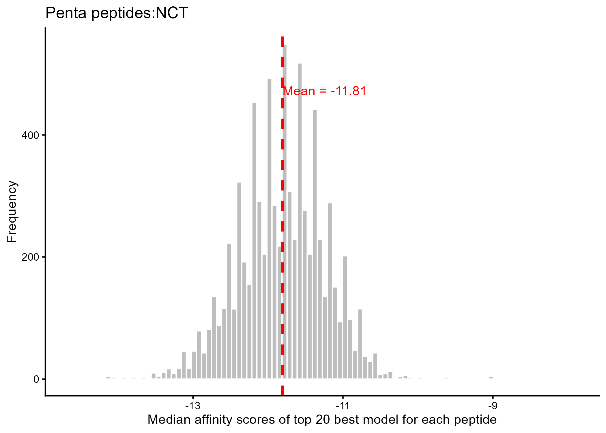

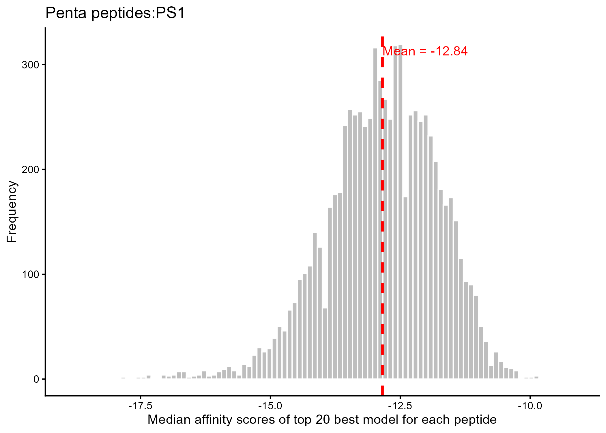
**

**
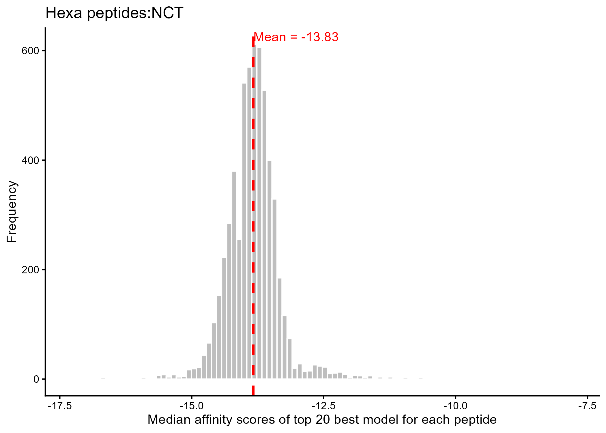

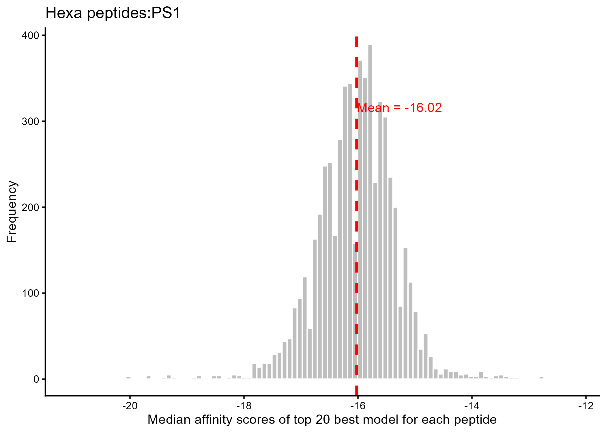
**

**
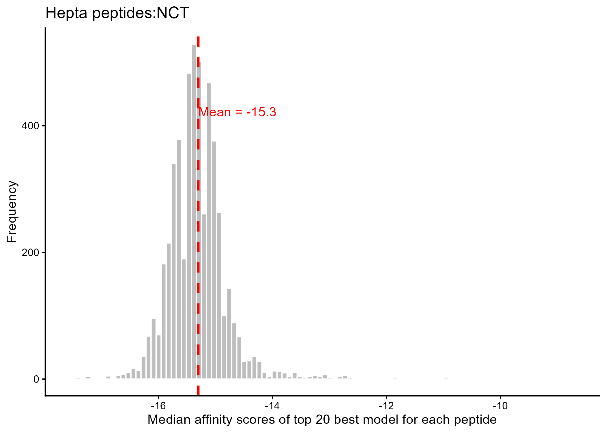

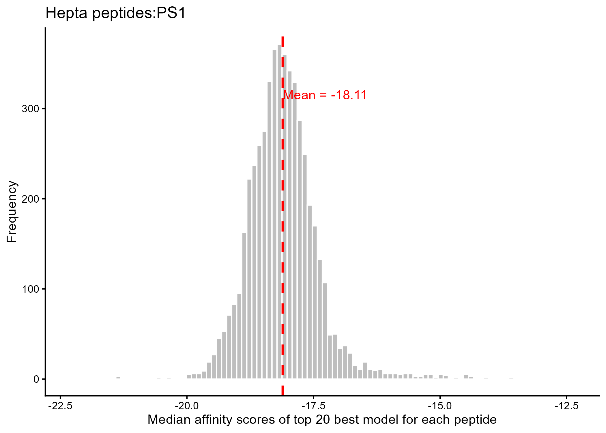
**

**
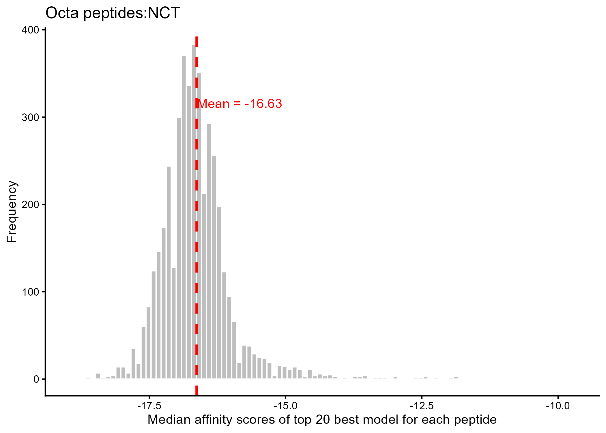

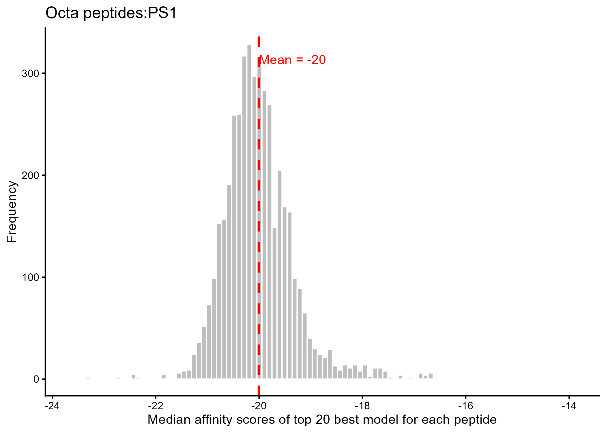
**

**
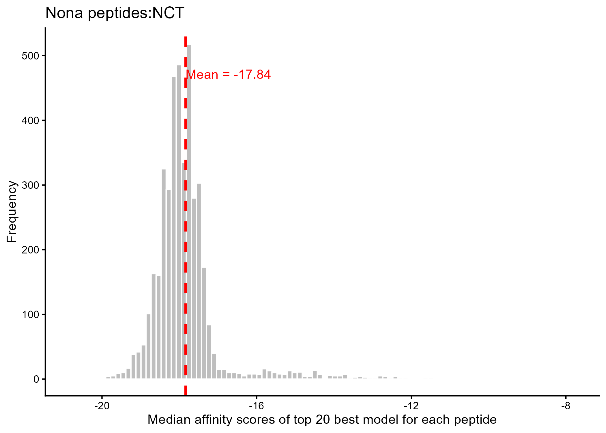

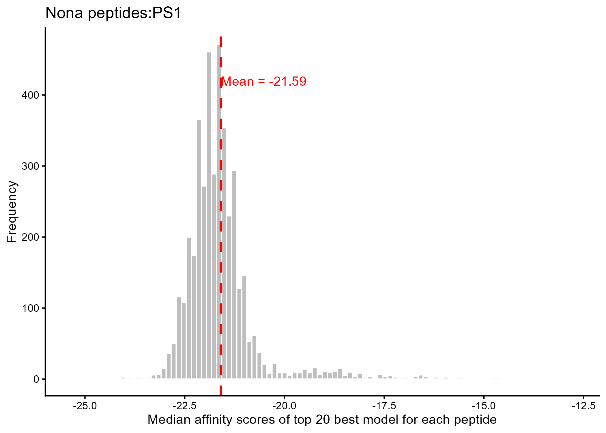
**

**
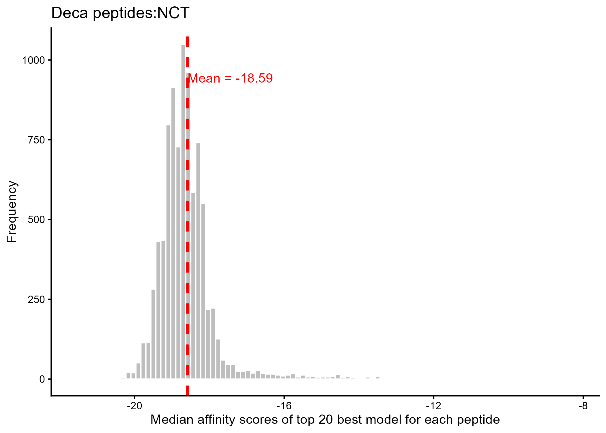

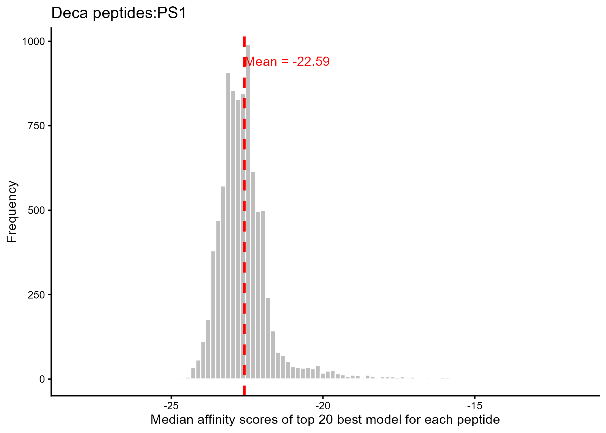
**

**Figure S1** **S1** Distribution of energies according to molecular docking results of peptide sets with NCT and PS1 binding sites of APH1. Energy values are provided as the median affinity scores of the top 20 models in each interaction.

The histograms (Figure S1) illustrate the frequency distribution of median binding energies. The red dashed line indicates the median affinity scores of the peptide sets. The distribution shows a near-normal profile centered around the mean, suggesting a consistent binding affinity across the generated peptide library, with a few outliers exhibiting superior -more negative- binding energies. When the overall landscape is examined, binding affinity scores have increased significantly in the iterative derivation of the peptide library. This systematic enrichment of the peptide population with higher affinity scores demonstrates that each iteration of library design narrows the virtual screening toward stronger candidates. The increase in the frequency of peptides showing favourable affinity scores in subsequent generations supports the structure-guided design strategy in identifying potential lead candidates with enhanced theoretical binding strengths.

**Table S1** Distribution by length of peptides with favorable physicochemical properties

| **Length** | 5 | 6 | 7 | 8 | 9 | 10 |
| --- | --- | --- | --- | --- | --- | --- |
| **Peptide count** | 742 | 765 | 364 | 106 | 27 | 116 |
